# Mapping HIV clustering: a strategy for identifying populations at high risk of HIV infection in sub-Saharan Africa

**DOI:** 10.1186/1476-072X-12-28

**Published:** 2013-05-22

**Authors:** Diego F Cuadros, Susanne F Awad, Laith J Abu-Raddad

**Affiliations:** 1 Infectious Disease Epidemiology Group, Weill Cornell Medical College – Qatar, Qatar Foundation - Education City, P.O. Box 24144, Doha, Qatar; 2 Department of Public Health, Weill Cornell Medical College, Cornell University, New York, NY, USA; 3 Vaccine and Infectious Disease Division, Fred Hutchinson Cancer Research Center, Seattle, WA, USA

**Keywords:** HIV, Spatial epidemiology, Disease mapping, Sub-Saharan Africa, Mathematical modeling

## Abstract

**Background:**

The geographical structure of an epidemic is ultimately a consequence of the drivers of the epidemic and the population susceptible to the infection. The ‘know your epidemic’ concept recognizes this geographical feature as a key element for identifying populations at higher risk of HIV infection where prevention interventions should be targeted. In an effort to clarify specific drivers of HIV transmission and identify priority populations for HIV prevention interventions, we conducted a comprehensive mapping of the spatial distribution of HIV infection across sub-Saharan Africa (SSA).

**Methods:**

The main source of data for our study was the Demographic and Health Survey conducted in 20 countries from SSA. We identified and compared spatial clusters with high and low numbers of HIV infections in each country using Kulldorff spatial scan test. The test locates areas with higher and lower numbers of HIV infections than expected under spatial randomness. For each identified cluster, a likelihood ratio test was computed. A *P*-value was determined through Monte Carlo simulations to evaluate the statistical significance of each cluster.

**Results:**

Our results suggest stark geographic variations in HIV transmission patterns within and across countries of SSA. About 14% of the population in SSA is located in areas of intense HIV epidemics. Meanwhile, another 16% of the population is located in areas of low HIV prevalence, where some behavioral or biological protective factors appear to have slowed HIV transmission.

**Conclusions:**

Our study provides direct evidence for strong geographic clustering of HIV infection across SSA. This striking pattern of heterogeneity at the micro-geographical scale might reflect the fact that most HIV epidemics in the general population in SSA are not far from their epidemic threshold. Our findings identify priority geographic areas for HIV programming, and support the need for spatially targeted interventions in order to maximize the impact on the epidemic in SSA.

## Background

Sub-Saharan Africa (SSA) has by far the largest HIV epidemic in the world, with 68% of the global HIV disease burden and 1.9 million new infections in 2010 [1]. The complex epidemiological context of this infection has prevented to date a clear identification of the specific drivers that have led to such large general population HIV epidemics in SSA, and nowhere else [2].

The ‘Know your epidemic, know your response’ is a Joint United Nation Programme on HIV/AIDS (UNAIDS) focused on HIV prevention [3], which has become one of the first calls to modify the current strategy by recognition of the fact that there is not a single global HIV epidemic. This conceptual approach emphasizes the necessity to identify populations at higher risk of HIV infection, and to implement an effective prevention strategy by acknowledgement of the substantial variation in which HIV spreads through populations and communities [4,5].

This strategy also highlights the significant role that geographical space plays in the identification of populations at higher risk. This fundamental characteristic of an epidemic, however, has been poorly explored in the context of HIV. Measures of disease occurrence are frequently available only by large geographical administrative units. These large scales (national or regional) could hide the natural scale of the HIV transmission process.

To overcome the current gap of knowledge about the spatial structure of the HIV epidemic, particularly in SSA, we explored and described the geographical heterogeneity of the HIV epidemic in SSA. We aimed to identify geographical settings where the risk of HIV infection is higher or lower. Studying the epidemics at their “microscopic” scale, that is through spatial mapping of the clustering of HIV infection at the local level, may provide a fresh look into the dynamics of these epidemics [6,7], and informed insights about their drivers in this part of the globe.

## Methods

### Data sources

The main source of data for our study were the Demographic and Health Survey (DHS) [8] databases. Countries were included for analysis based upon the availability of DHS HIV serological biomarker survey and the geographical coordinates of each survey data point. For each country, we only considered the most recent DHS where HIV data were collected. As a result, a total of 20 countries in SSA were included: Burkina Faso (2010), Burundi (2010), Cameroon (2004), Congo Democratic Republic (2007), Ethiopia (2011), Ghana (2003), Guinea (2005), Kenya (2008–2009), Lesotho (2009), Liberia (2007), Malawi (2010), Mali (2006), Mozambique (2009), Rwanda (2010), Senegal (2010–2011), Swaziland (2006–2007), Sierra Leone (2008), Tanzania (2007–2008), Zambia (2007), and Zimbabwe (2010–2011).

### Spatial clustering detection

We identified the spatial clusters with high and low numbers of HIV infections in each country through a Kulldorff spatial scan statistics analysis [9]. This methodology has become the most widely used test for clustering detection in epidemiology [10-12], and its efficiency and accuracy has been well documented [13,14]. A spatial scan statistics is a cluster detection test able to find the location of areas with higher or lower numbers of cases (for instance HIV infections) than expected under spatial randomness, and then evaluate their statistical significance by gradually scanning a circular window that spans the study region. The radius of the circle is changed continuously so that it can take any value from 0 up to a pre-specified maximum value. A maximum circular window of 100 Km radius was used for scanning potential clusters with high or low numbers of HIV infections.

### Statistical analysis

For each potential cluster, a likelihood ratio test was computed assuming that the number of HIV infections in each circular window is an independent Bernoulli random variable. The numbers of observed and expected HIV infections within and outside the circular window were then compared with the likelihood *L*_*0*_ under the null hypothesis of spatial randomness. The circular windows with the highest likelihood ratio values were identified as potential clusters. An associated *P*-value of the statistics was then determined through Monte Carlo simulations and used to evaluate whether HIV infections are randomly distributed in space or not.

### Cluster characterization

After a cluster was identified, the strength of the clustering was estimated using the relative risk (RR) of HIV infection within the cluster versus outside the cluster. The fraction of the population, and HIV prevalence were also estimated for each cluster. Furthermore, the general RR of HIV infection for all individuals belonging to (high or low) clusters was also estimated by combining all (high or low) clusters identified in a particular country. All geographic information system (GIS) analysis and cartographic displays were performed with the software ArcGIS version 9.2 [15].

### Mathematical modeling

A deterministic compartmental mathematical model was constructed based on extension of earlier models [16-18] to describe the heterosexual transmission of HIV in a given population [18]. The model consists of a system of coupled nonlinear differential equations, and stratifies the population according to HIV status, stage of infection and sexual risk group. Our model incorporates 10 sexual risk groups in the population, starting from lower to higher levels of sexual risk behavior. The level of sexual risk behavior was parameterized by the effective partnership change rate in each risk group, and in essence it is a measure of the risk of exposure to the HIV infection. Further details about the model structure can be found in the Additional file 1.

## Results

**Figure 1:**
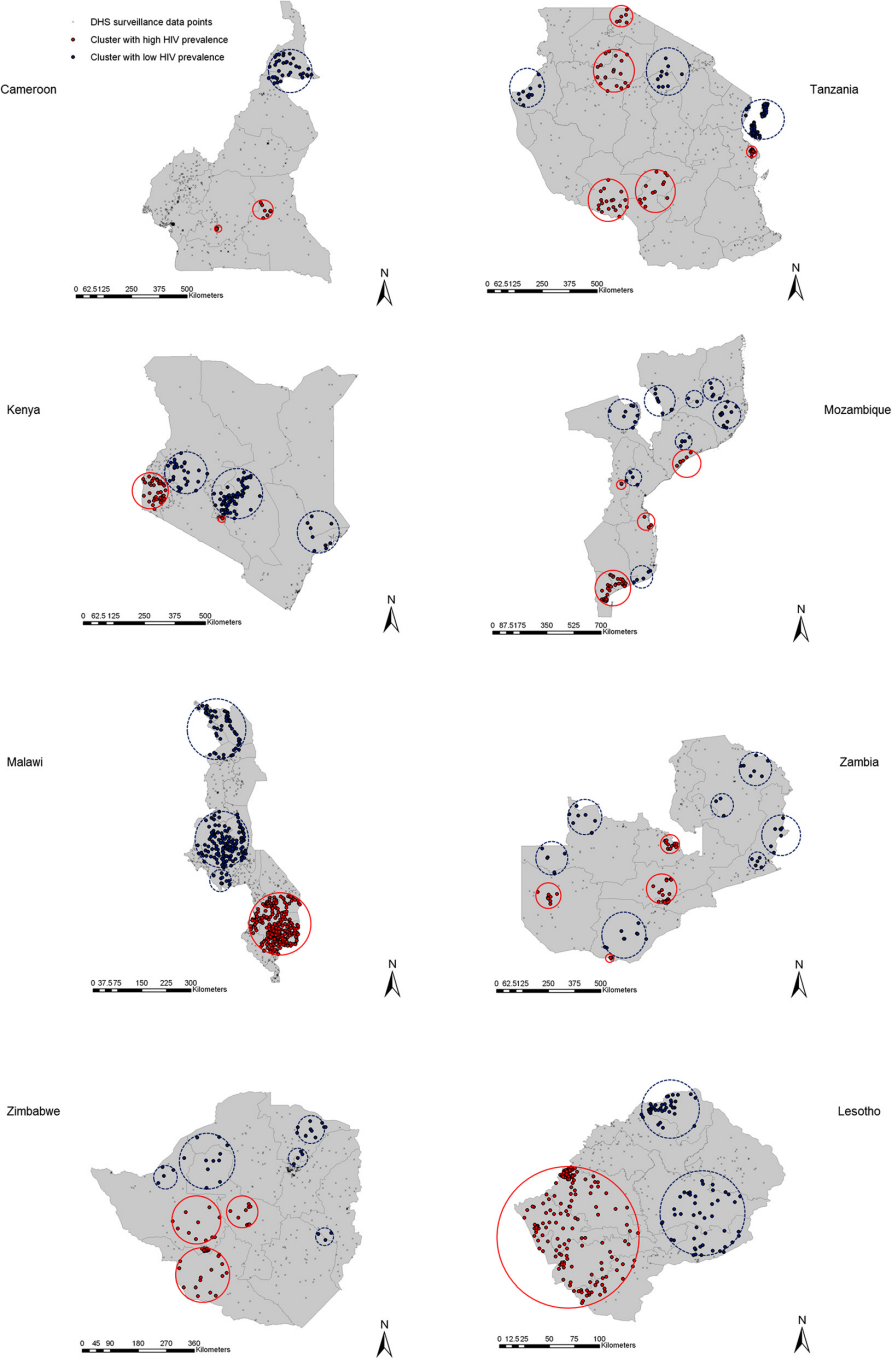
**Spatial distribution of the clusters with high and low HIV prevalence in countries with national HIV prevalence higher than 4%.** Geographical localization of the clusters with high (red-solid circles) and low (blue-dashed circles) HIV prevalence.

**Figure 2:**
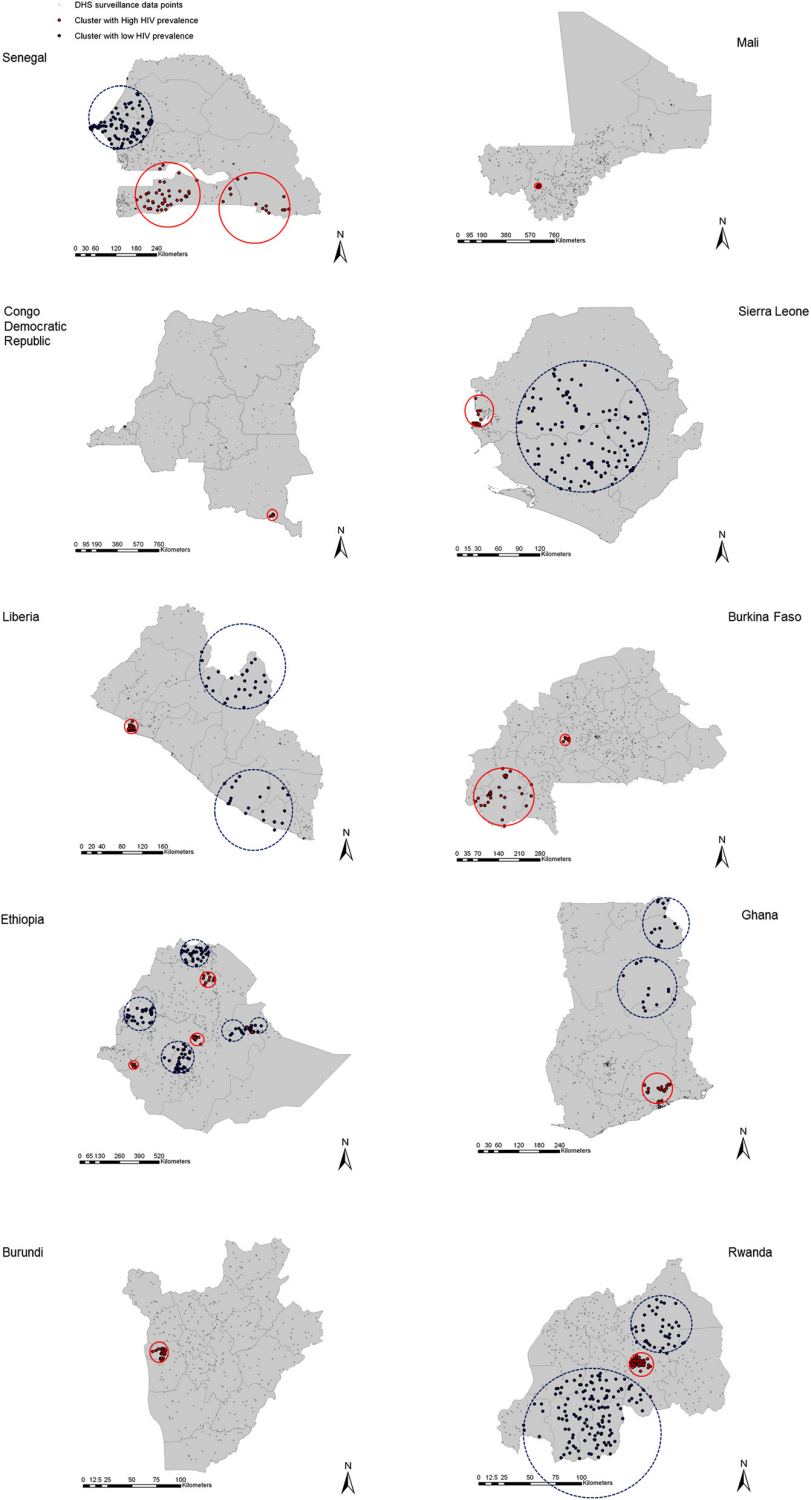
**Spatial distribution of the clusters with high and low HIV prevalence in countries with national HIV prevalence lower than 4%.** Geographical localization of the clusters with high (red-solid circles) and low (blue-dashed circles) HIV prevalence.

Our analysis identified 38 clusters with high HIV prevalence, and 45 clusters with low HIV prevalence. The locations of these clusters are illustrated in Figure 1 for countries with HIV prevalence larger than 4%, and in Figure 2 for countries with HIV prevalence lower than 4%.

From the 20 countries included in the study only two, Guinea and Swaziland, did not have clusters with either high or low HIV prevalence, possibly as a consequence of the small geographic size of these countries. Clusters with only high HIV prevalence were identified in Burkina Faso, Burundi, Democratic Republic of Congo and Mali. Clusters with high and low HIV prevalence were identified in the remaining 14 countries. General description of the clusters for each country is summarized in Table 1, and a more detailed characterization of each cluster is included in Additional file 1: Table S1.

**Table 1:** General description of the clusters with high and low HIV prevalence identified in the 20 countries included in the study

**Country**	**National HIV prevalence**	**Clusters with high HIV prevalence**				**Clusters with low HIV prevalence**			
		**Number of clusters**	**Fraction of the population (%)**	**HIV prevalence range (%)**	Strength of the clustering^*^ range	**Number of clusters**	**Fraction of the population (%)**	**HIV prevalence range (%)**	Strength of the clustering^*^ range
Senegal	0.75	2	14.88	1.94 – 4.35	3.33 – 6.69	1	32.80	0.24	0.24
Mali	1.11	1	11.92	2.55	2.77	0	0	–	–
Congo D. R.	1.39	1	2.43	5.00	3.86	0	0	–	–
Sierra Leone	1.49	1	8.39	5.03	4.31	1	41.00	0.70	0.34
Guinea	1.55	0	0	–	–	0	0	–	–
Liberia	1.59	1	18.07	3.31	2.74	2	17.14	0 – 0.08	0 – 0.004
Burkina Faso	1.69	2	15.91	3.61 – 7.73	2.59 – 4.99	0	0	–	–
Ethiopia	1.80	5	16.12	4.86 – 8.23	3.19 – 4.85	5	22.05	0 – 0.50	0 – 0.27
Ghana	1.86	1	3.88	5.57	3.25	2	10.19	0	0
Burundi	1.88	1	12.74	4.16	2.65	0	0	–	–
Rwanda	3.17	1	12.04	8.24	3.32	2	34.02	1.04 – 1.92	0.31 – 0.53
Tanzania	4.02	5	14.30	8.33 – 17.70	2.18 – 4.41	3	33.9	0 – 0.77	0 – 0.14
Cameroon	5.44	2	5.53	12.13 – 18.18	2.3 – 3.44	1	8.67	1.47	0.25
Kenya	6.80	2	11.09	21.61 – 29.73	4.26 – 4.28	3	26.33	0 – 2.6	0 – 0.52
Mozambique	8.66	4	23.9	15.05 – 22.01	2.07 – 2.21	8	18.42	0 – 2.69	0 – 0.31
Malawi	10.38	1	34.98	15.38	2.11	3	28.79	0 – 4.95	0 – 0.47
Zambia	14.62	4	22.61	22.20 – 25.21	1.70 – 1.74	7	13.89	0.99 – 8.17	0.07 0.55
Zimbabwe	16.49	3	14.46	20.76 – 30.75	1.29 – 1.91	5	10.78	1.90 – 9.94	0.11 – 0.59
Swaziland	19.10	0	0	–	–	0	0	–	–
Lesotho	22.22	1	40.71	25.49	1.28	2	21.20	14.19 – 16.37	0.31 – 0.71

*Strength of the clustering estimated as the relative risk of HIV infection within the cluster versus outside the cluster.

HIV prevalence within the clusters with high prevalence ranged from 1.9% in a cluster in Senegal (RR = 3.33, *P* < 0.001) to 30.8% in a cluster in Zimbabwe (RR = 1.91, *P* < 0.001), with a median of 11.5%. Likewise, HIV prevalence within the clusters with low HIV prevalence ranged from 16.4% in a cluster in Lesotho (RR = 0.71, *P* = 0.034) to clusters with 0% HIV prevalence in Liberia, Ethiopia, Ghana, Tanzania, Kenya, Mozambique, and Malawi, with a median of 1.7%.

The fraction of the population within clusters with high HIV prevalence had a median of 14.4%, and this fraction increased with the national HIV prevalence (*P* = 0.005). This association, however, did not explain most of the variation (adjusted R-squared = 0.41). The fraction of the population within clusters with low HIV prevalence had a median of 15.5%. However, we found no statistically significant trend in the association between the fraction of the population within clusters with low HIV prevalence and the national HIV prevalence (*P* = 0.73).

**Figure 3:**
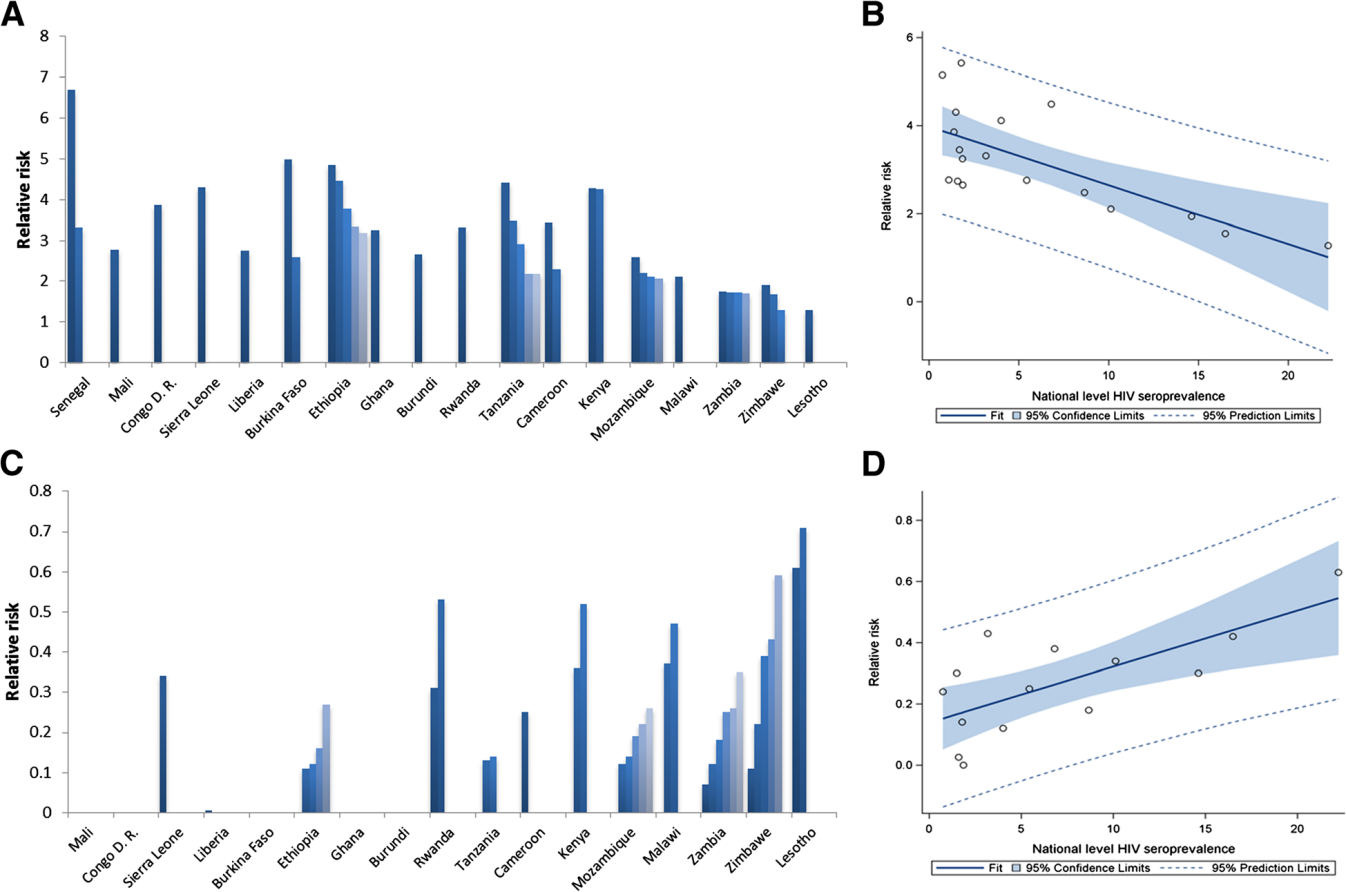
**Strength of HIV clustering. (A)** Relative risk of HIV infection in clusters with high HIV prevalence (each bar represents the relative risk in a single cluster). **(B)** Association between relative risk of HIV infection in clusters with high HIV prevalence and the national HIV prevalence. **(C)** Relative risk of HIV infection in clusters with low HIV prevalence (each bar represents the relative risk in a single cluster). **(D)** Association between relative risk of HIV infection in clusters with low HIV prevalence and the national HIV prevalence. Countries are shown in order of increasing national HIV prevalence.

The RR of HIV infection for individuals within clusters with high HIV prevalence was negatively associated with the national HIV prevalence (*P* < 0.001) (Figure 3B). Conversely, the RR of HIV infection for individuals within clusters with low HIV prevalence was positively associated with the national HIV prevalence (*P* < 0.001) (Figure 3D). The HIV prevalence in clusters with both high and low HIV prevalence was positively associated with the national HIV prevalence (*P* < 0.001).

**Figure 4:**
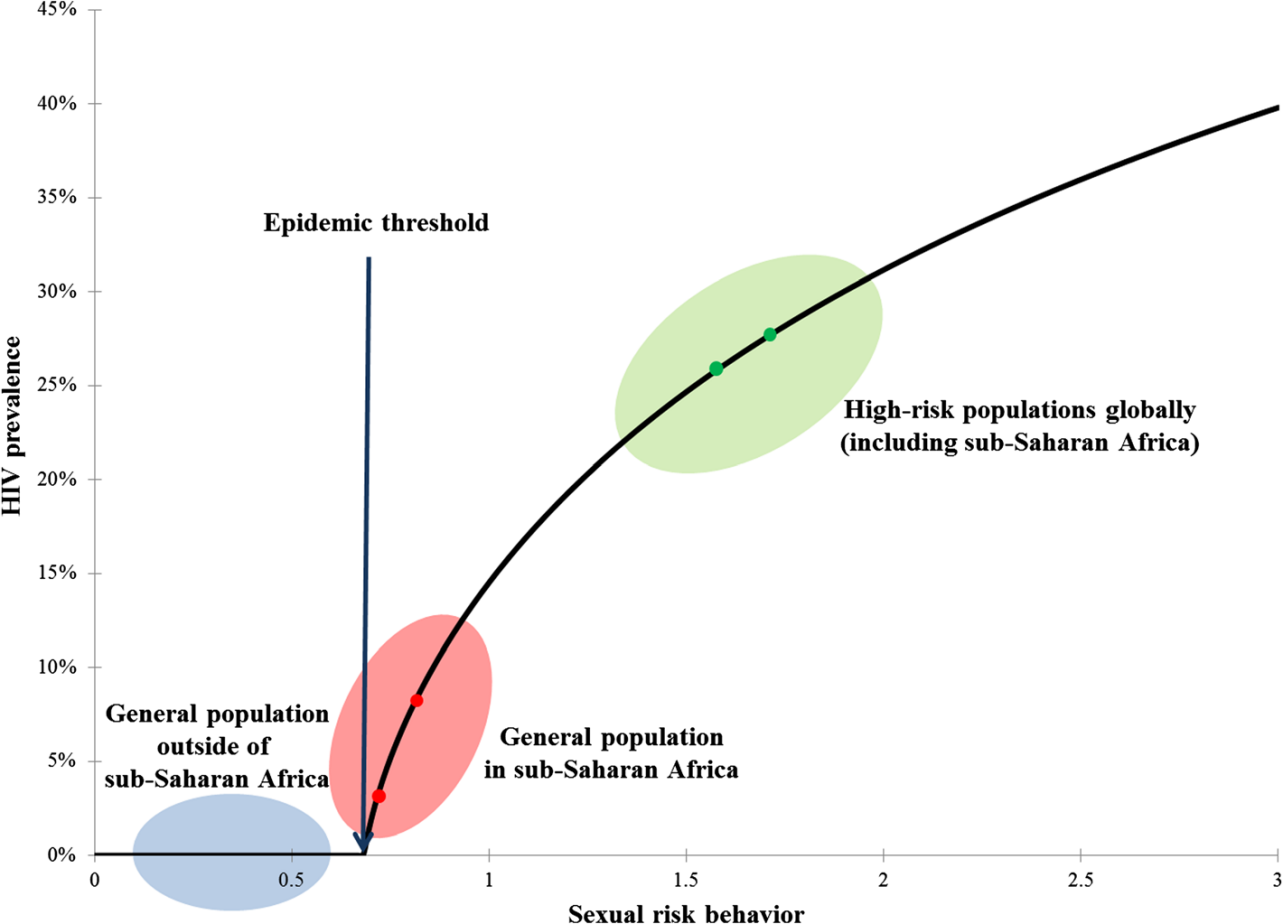
**Generic relationship between sexual risk behavior (or HIV risk of exposure) and HIV prevalence in an HIV epidemic.** Three dynamical regions can be discerned: 1) Below the epidemic threshold (blue zone) representing the HIV dynamics among the general population outside of sub-Saharan Africa (SSA). 2) Just above the epidemic threshold (red zone) representing the hypothesized HIV dynamics among much of the general population of SSA. 3) Well above the epidemic threshold (green zone) representing the HIV dynamics among high-risk populations globally (including SSA). These results were generated using a conventional deterministic HIV epidemic model [16-18].

The mathematical model constructed to assess the behavior of the epidemic around the epidemic threshold showed that a modest change of 10% in sexual risk behavior (illustrated by the red dots in Figure 4), corresponding for example to a 10% increase in the number of sexual partners per year (or a 20% reduction in male circumcision coverage among adult males), leads to 250% increase in HIV prevalence. Beyond the region of epidemic threshold, the same increase in sexual risk behavior (illustrated by the green dots in Figure 4) leads to only 8% increase in HIV prevalence, a factor about 30 times smaller than the increase near the epidemic threshold.

## Discussion

The results of our analysis indicate stark geographical variation in HIV prevalence in most of the countries. The observed spatial variation in HIV prevalence highlights a clustered HIV transmission across SSA within micro-epidemics of different scales. The map of HIV clustering reflects a landscape with ‘valleys’ (areas with high HIV prevalence), ‘dams’ (areas where HIV found barriers to propagate efficiently), and ‘islands’ (small isolated areas with characteristically either very high or very low HIV prevalence).

Our results indicate that only ~14% of the population across the countries resides within clusters of high HIV prevalence. The strength of the clustering tended to be higher in countries with low national HIV prevalence. For instance, the strongest clustering (highest RR) is found in a cluster in Senegal (RR = 6.69, HIV prevalence = 4.3%); the country with the lowest national HIV prevalence (0.7%). Our study revealed similar settings with localized epidemics at high HIV prevalence hidden in a map of low national HIV prevalence, such as in Burkina Faso, Congo, Sierra Leone and Ethiopia.

The strength of the clustering was smaller in countries with high national HIV prevalence, indicative of more diffusive epidemics. For instance, in Zambia and Lesotho, the strength of the clustering was fairly small (RR = 1.74, and RR = 1.28, respectively). In Swaziland, no clusters with high HIV prevalence were identified. This result underlines how the HIV epidemics in these high prevalence countries had percolated throughout much of the demography and geography of these countries.

We also identified clusters with low HIV prevalence in most of the countries included in our study. These clusters appear to reflect ‘dams’ where some behavioral or biological protective factors appear to have slowed HIV transmission in such populations, in contrast to their neighboring populations. In fact, we identified settings with very low HIV prevalence even in countries with substantial HIV epidemics such as in Tanzania, Kenya and Malawi.

The topography of this infection poses a question about the drivers of such stark heterogeneities even at micro-geographic scales within countries. Male circumcision [19], the presence of other sexually transmitted infections (STIs) [20], tropical co-infections increasing HIV viral load [17], hormonal contraception [21], viral factors [22], and host genetics and immunology [22] vary across SSA, and are believed to influence HIV transmission risk. Behavioral factors such as concurrency [23], number of sexual partners [24], commercial sex [25], and coital frequency [26] appear also to vary across SSA, and may contribute to explaining the heterogeneities in prevalence. Preliminary statistical analyses of the DHS databases (not shown) indicated that it is challenging, if not a formidable task, to disentangle the contribution of the different factors in the clustering of the infection. This is a consequence of the complex array of independent variables to consider, and also because of the population sizes of the clusters’ sub-samples which are not large enough to power meaningful multivariate regression analyses. Nevertheless, these preliminary analyses suggest that the scale and distribution of the differences in the biological and behavioral factors, within versus outside the clusters, may not be sufficient to explain the observed sharp contours in the topography of HIV infection at the local level. We suspect that there is an additional dynamical factor that has strongly influenced the local ecology of this infection even when the differences in the biological and behavioral factors may not have been markedly large.

We hypothesize that the HIV epidemic among the general population in much of SSA is not far from its epidemic (or sustainability) threshold. A generic feature of an infection epidemic is that near the epidemic threshold, the prevalence depends non-linearly on the determinants of infection transmission, and that small changes in the epidemiological context can drive much larger changes in the prevalence of the infection [27]. Figure 4 illustrates this dynamical effect for the case of HIV infection. As can be seen in the figure, modest changes in sexual risk behavior near the epidemic threshold could generate a substantial increase in HIV prevalence. Conversely, beyond the region of epidemic threshold, the same increase in sexual risk behavior could generate only a modest increase in HIV prevalence.

Accordingly, we hypothesize that an essential driver of the stark variability in HIV infection transmission in SSA is that the epidemiology of this infection is not far from its epidemic threshold in the general population outside of conventional high-risk groups. The conspicuously large clustering of HIV infection may not strictly reflect conspicuously large variations in sexual risk behavior or the presence (or absence) of specific biological co-factors in HIV transmission. The variability in sexual risk behavior or biological co-factors within the population has driven a much larger variability in HIV prevalence, thanks to the non-linear epidemic dynamics near the infection sustainability threshold. This hypothesis may also contribute to explaining the global variability in HIV infectious spread where only in SSA massive general-population HIV epidemics have occurred. In SSA, but nowhere else, the epidemiology of HIV infection has crossed, though not by far, the epidemic threshold of sustainability in the general population (Figure 4). That was enough to spark localized epidemics in the general population; and these epidemics, not far above the sustainability threshold, exhibited consequently high diversity in size at the micro-geographic scale (Figures 1 and 2).

Several study limitations could have affected our results. First, the selection of the DHS round for the different countries was constrained by the availability of HIV biomarker information and geographical coordinates of each survey data point at any particular DHS round. This limited our ability to consider more countries in SSA for analysis with more recent DHS rounds. Small clusters of HIV infection could have been missed if there is not enough sampling within their geographic setting. Given the multiple logistical difficulties in conducting the DHS, some of our measures could have been influenced by inherent biases in the data such as the variability in response rates to HIV testing [28,29].

Mobile individuals and high-risk subpopulations such as female sex workers, injecting drug users, and men who have sex with men, may have been undersampled by the DHS. Clusters of HIV infection among such subpopulations may have been missed in our analysis. It is not clear though whether undersampling of such populations could necessarily affect our findings or not. Epidemics among high-risk subpopulations should lead to some infection onward transmission such as among spouses and clients of female sex workers which are less likely to be undersampled in the DHS. Lastly, due to the cross-sectional nature of the data used in this study, some of the clusters identified here could reflect epidemics at different stages, rather than genuine differences in epidemic sizes. The HIV epidemic in SSA, however, is in a mature stage [25,30], and therefore this potential limitation is probably not influencing our results. Moreover, we analyzed the clustering of the infection in four countries that had more than one DHS serological biomarker survey at different years, and no consequential changes in the distribution of the clusters were observed (not shown).

## Conclusions

In sum, our study provides evidence for a striking geographic clustering of HIV infection across SSA. The exact drivers of such rich and complex infection topography are not well understood. However, the clustering possibly reflects differences in specific behavioral and biological factors between sub-populations that have been amplified as larger differences in HIV prevalence, as a consequence of the infection epidemiology being not far from its epidemic threshold. Our findings support the need for spatially-targeted prevention strategies in SSA, and our results delineate the map of the high disease-burden areas. If indeed HIV epidemiology in SSA is not far from its epidemic threshold in the general population, this would indicate that even modest intervention-driven changes in risk behavior, or risk of HIV acquisition, through behavioral or biomedical interventions, may have considerable impact in reducing the sizes of the African epidemics. The recently observed rapid declines in HIV prevalence in SSA [1,31,32] may suggest that this transition is already taking place.

## Abbreviations

SSA: Sub-Saharan Africa; UNAIDS: Joint United Nations Programme on HIV/AIDS; DHS: Demographic and Health Survey; RR: Relative risk; GIS: Geographic information system; STI: Sexually transmitted infection.

## Competing interest

The authors declare that they have no competing interest.

## Authors’ contributions

DFC conceived the study and its design, conducted most of the statistical and mathematical modeling analyses, and wrote the first draft of the paper. SFA contributed to the conduct of the mathematical modeling analyses and interpretation of the results. LJA-R contributed to study conception and design, conduct of the statistical and mathematical modeling analyses, interpretation of the results, and writing of the manuscript. All authors read and approved the final manuscript.

## Supplementary Material

Additional file 1 **Supplementary methods. Table S1.** Characterization of the clusters with high and low HIV prevalence identified in the 20 countries included in the study. **Table S2.** Model assumptions in terms of parameter values. Click here for file
