# Supplementary material for: Mapping HIV clustering: a strategy for identifying populations at high risk of HIV infection in sub-Saharan Africa
Source: Int J Health Geogr. 2013 May 22;12:28. doi: 10.1186/1476-072X-12-28 (PMC3669110; doi:10.1186/1476-072X-12-28)
Supplement: Additional file 1 — Supplementary methods. Table S1. Characterization of the clusters with high and low HIV prevalence identified in the 20 countries included in the study. Table S2. Model assumptions in terms of parameter values. [file 1476-072X-12-28-S1.pdf]

Supplementary Materials

Mapping HIV clustering: A strategy for identifying populations at high risk of HIV infection in sub-Saharan Africa

Diego F. Cuadros[1, 2*], Susanne F. Awad[1] and Laith J. Abu-Raddad[1, 2, 3]

[1]*Infectious Disease Epidemiology Group, Weill Cornell Medical College - Qatar, Cornell University, Qatar Foundation - Education City, Doha, Qatar*

[2]*Department of Public Health, Weill Cornell Medical College, Cornell University, New York 10065-4896, USA*

[3]*Vaccine and Infectious Disease Division, Fred Hutchinson Cancer Research Center, Seattle, Washington 98109, USA*

*To whom correspondence should be addressed. E-mail: dfc2002@qatar-med.cornell.edu

Supplementary methods

Mathematical model structure

We used a deterministic compartmental mathematical model of the heterosexual transmission of HIV in a population to illustrate the influence of the level of sexual risk behavior on the dynamics of HIV prevalence near the epidemic threshold. The model was developed to study HIV epidemiology and sexual risk behavior in sub-Saharan Africa [1], and was constructed based on extension of earlier models [2, 3]. The model stratifies the sexually active population into compartments according to HIV status, stage of infection, and sexual risk behavior.

The model was expressed in terms of a system of coupled nonlinear differential equations for each risk group as following:

$$\frac{dS_i}{dt} = \mu N_{0,i} - \mu S_i - \Lambda_i S_i \qquad \text{(S1)}$$

$$\frac{dI_{i,1}}{dt} = \Lambda_i S_i - \mu I_{i,1} - \omega_1 I_{i,1} \qquad \text{(S2)}$$

$$\frac{dI_{i,2}}{dt} = \omega_1 I_{i,1} - \mu I_{i,3} - \omega_2 I_{i,2} \qquad \text{(S3)}$$

$$\frac{dI_{i,3}}{dt} = \omega_2 I_{i,2} - \mu I_{i,3} - \omega_3 I_{i,3}\,. \qquad \text{(S4)}$$

To introduce heterogeneity in sexual risk behavior, we stratified the population into 10 sexual risk groups, defined with the index i ($i = 1, 2, ..., 10$ representing low to high risk groups). S_i is the HIV susceptible population in the i–risk group, and $I_{i,\beta}$ is the HIV infected population in the i–risk group. The index $\beta = 1, 2, 3$ represents the acute, latent, and advanced stages of the infection, respectively. $N_{0,i}$ is the initial population size of each i-risk group. μ stands for the natural mortality/leaving the sexually active population rate. The rate of progression from one HIV stage to the next is described by

ω_1 and ω_2, while ω_3 is the rate of HIV/AIDS disease mortality. Λ_i calculates the HIV force of infection experienced by the S_i susceptible population. Λ_i is given by:

$$\Lambda_i = \rho_{S_i} \sum_{j=1}^{10} \sum_{\beta=1}^{3} t_{I_{j,\beta} \to S_i} \mathcal{G}_{i,j} \frac{\rho_{I_{j,\beta}} I_{j,\beta}}{\rho_{S_j} S_j + \sum_{\beta=1,2,3} \rho_{I_{j,\beta}} I_{j,\beta}}, \quad \text{(S5)}$$

where ρ_{X_i} describes the level of sexual risk behavior for each population variable X_i. ρ_{X_i} summarizes behavioral characteristics such as the rate of new sexual partners an individual in a specified risk group acquires. But it also characterizes effectively other factors that enhance the risk of exposure to the infection such as concurrency and clustering within sexual networks [2, 4-8], and variability in sexual risk behavior in the population [9]. Since the precise nature of sexual behavior and sexual networks in sub-Saharan Africa is not sufficiently understood, and varies within and across communities [10, 11], ρ_{X_i} is a summary measure of the population-specific level of sexual risk behavior, and captures the distribution and strength of the risk of exposure to HIV infection.

The form of ρ_{X_i} distribution across different risk groups was defined through a power law function as

$$\rho_{X_i} = Ci^{\alpha}. \quad \text{(S9)}$$

This form is motivated by simulations using an individual-based network model developed to explore the diversity in the level of sexual risk behavior [12], by analyses of the architecture of complex weighted networks [13, 14], and by an analysis of the average separation between individuals in a network or a sub-network [15, 16]. Here C is a constant determined by the average risk behavior and α is the exponent parameter that determines the level of variability in the effective sexual partner acquisition rate [12].

The parameter $t_{I_{j,\beta}\to S_i}$ in equation S5 defines HIV transmission probability per partnership between an individual of the susceptible population, S_i, and an individual of the HIV infected population $I_{j,\beta}$. It is expressed in terms of HIV transmission probability per coital act per HIV stage in this partnership ($p^{HIV}_{I_{j,\beta}\to S_i}$), the frequency of coital acts per unit time in this partnership ($n_{I_{j,\beta}\leftrightarrow S_i}$), and the duration ($\tau_{I_{j,\beta}\leftrightarrow S_i}$) of this partnership:

$$t_{I_{j,\beta}\to S_i} = 1-\left(1-p^{HIV}_{I_{j,\beta}\to S_i)}\right)^{n_{I_{j,\beta}\leftrightarrow S_i}\tau_{I_{j,\beta}\leftrightarrow S_i}} . \qquad \text{(S6)}$$

The mixing among the different risk groups is dictated by the sexual-mixing matrix $\mathcal{G}_{i,j}$. This matrix provides the probability that an individual in risk group i would choose a partner in risk group j. It is given by

$$\mathcal{G}_{i,j} = e\delta_{i,j} + (1-e)\frac{\rho_{S_j} S_j + \sum_{\beta=1,2,3}\rho_{Y_{\alpha',j}} I_{\beta,j}}{\sum_{k=1,2,3,4}\left(\rho_{S_k} S_k + \sum_{\beta=1,2,3}\rho_{I_{\beta,k}} I_{\beta,k}\right)} . \qquad \text{(S7)}$$

Here, $\delta_{i,j}$ is the identity matrix and the parameter $e\in[0,1]$ measures the degree of assortativeness in the mixing. At the extreme $e=0$, the mixing is proportionate (choosing partners with no preferential bias based on the kind of risk group) while at the other extreme $e=1$, the mixing is fully assortative as individuals choose partners only from within their own risk group [17].

Sexual risk behavior

The sexually active population was stratified into a number of risk groups where the proportion of the population initially in each risk group i was determined using a gamma distribution

$$p(i) = \frac{1}{b^a\Gamma(a)} i^{a-1} e^{\frac{-i}{b}} \qquad \text{(S8)}$$

The gamma distribution is motivated by the degree distribution of the number of sexual partners as identified empirically in studies in sub-Saharan Africa [18-21]. Here a is the shape parameter determined through normalization of the distribution, and b is the scale parameter in the gamma distribution of the population size across the risk groups [18].

Parameter values

The parameters of the model were derived using the best available empirical data on HIV epidemiology and natural history, and are listed in Table S2 along with their references. We based HIV transmission probability per coital per each of HIV stages on recent re-analyses of the Rakai Study data [2, 22-24]. The frequency of coital acts per each stage of infection was based on the measurements of Wawer *et al* [24].

The durations of the acute, latent, and advanced stages were assumed to be 49 days (acute), 9 years (latent), and 2 years (advanced). These choices were based on recent compilation of data by UNAIDS indicating that the average duration from HIV acquisition to death, in absence of antiretroviral therapy, is about 11 years [25, 26], and based on the classification in Wawer *et al* [24], re-analysis of the Rakai data for acute infection [22], and measured time from seroconversion to death in several cohort studies [27, 28]. The duration of the sexual activity lifespan is 35 years per definition of the sexually active population in the literature, and conventional guidelines (15-49 years age group) [2, 29, 30].

As for the parameters of sexual risk behavior, the degree of assortativeness (e) was fixed at 0.3; a representative value based on model calibration of the HIV epidemic in Kisumu, Kenya [2]. Meanwhile, the scale parameter in the gamma distribution of the population across the different risk groups (b) was fixed at 2, based on fitting empirical data of the degree distribution (number of sexual partners per year) [18]. The exponent parameter in the power law function of the distribution of sexual risk behavior (α) was fixed at 1.7 for

all countries, based on fitting the distribution of the clustering coefficient of all possible configurations in a sexual network [12].

Supplementary tables

Supplementary Table 1 - Characterization of the clusters with high and low HIV prevalence identified in the 20 countries included in the study

Country	Cluster	Sample size	Number of HIV infections	Relative risk (RR)	Radius of cluster (Km)	*P* value
**Senegal**	High 1	276	12	6.69	95.39	0.001
	High 2	1232	24	3.33	94.84	0.007
	Low 1	3330	8	0.24	92.33	0.002
**Mali**	High 1	1060	27	2.77	11.09	0.026
**Congo R. D.**	High 1	220	11	3.86	32.85	0.047
**Sierra Leone**	High 1	557	28	4.31	19.52	<0.001
	Low 1	2727	19	0.34	96.66	0.004
**Liberia**	High 1	2112	70	2.74	11.34	<0.001
	Low 1	1274	1	0.004	99.37	<0.001
	Low 2	729	0	0	81.51	0.001
**Burkina Faso**	High 1	168	12	4.99	12.89	0.006
	High 2	997	36	2.59	98.28	0.008
**Ethiopia**	High 1	425	35	4.85	1.98	<0.001
	High 2	618	46	4.45	4.44	<0.001
	High 3	304	20	3.77	18.96	0.002
	High 4	2862	139	3.34	46.22	<0.001
	High 5	395	22	3.19	43.17	0.011
	Low 1	483	0	0	88.76	0.044
	Low 2	940	2	0.11	48.74	0.007
	Low 3	1386	3	0.12	99.09	<0.001
	Low 4	1688	5	0.16	97.50	<0.001
	Low 5	1799	9	0.27	88.15	0.002
**Ghana**	High 1	377	21	3.25	42.13	0.009
	Low 1	594	0	0	83.53	0.002
	Low 2	397	0	0	84.98	0.045
**Burundi**	High 1	1131	47	2.65	13.22	<0.001
**Rwanda**	High 1	1626	134	3.32	10.51	<0.001
	Low 1	1155	12	0.31	29.89	0.003
	Low 2	3437	66	0.53	74.32	0.002
**Tanzania**	High 1	305	54	4.41	98.80	<0.001
	High 2	396	52	3.48	20.39	<0.001
	High 3	530	58	2.91	94.40	<0.001
	High 4	660	55	2.19	99.86	<0.001

	High 5	201	22	2.18	41.00	0.018
	Low 1	4387	34	0.14	99.6	<0.001
	Low 2	281	0	0	72.34	0.002
	Low 3	368	2	0.13	82.96	0.016
**Cameroon**	High 1	121	22	3.44	2.50	0.001
	High 2	239	29	2.3	40.84	0.045
	Low 1	883	13	0.25	99.93	<0.001
**Kenya**	High 1	694	150	4.28	67.85	<0.001
	High 2	37	11	4.26	5.52	0.014
	Low 1	174	0	0	93.75	0.002
	Low 2	500	13	0.36	85.99	0.028
	Low 3	1061	41	0.52	99.24	0.021
**Mozambique**	High 1	218	48	2.60	3.94	<0.001
	High 2	315	59	2.21	70.84	<0.001
	High 3	3235	487	2.11	97.07	<0.001
	High 4	221	39	2.07	72.64	0.014
	Low 1	101	0	0	60.08	0.017
	Low 2	286	3	0.12	75.43	<0.001
	Low 3	566	7	0.14	96.10	<0.001
	Low 4	473	8	0.19	95.12	<0.001
	Low 5	311	6	0.22	57.25	<0.001
	Low 6	732	17	0.26	99.80	<0.001
	Low 7	309	8	0.30	52.73	0.013
	Low 8	297	8	0.31	65.00	0.017
**Malawi**	High 1	4629	712	2.11	94.49	<0.001
	Low 1	156	0	0	32.57	<0.001
	Low 2	2524	107	0.37	87.93	<0.001
	Low 3	1131	56	0.47	93.41	<0.001
**Zambia**	High 1	599	133	1.74	40.88	<0.001
	High 2	1246	290	1.72	66.62	<0.001
	High 3	363	89	1.72	54.26	<0.001
	High 4	234	59	1.70	5.74	0.010
	Low 1	101	1	0.067	84.82	<0.001
	Low 2	113	2	0.12	84.52	0.002
	Low 3	152	4	0.18	91.30	<0.001
	Low 4	191	7	0.25	81.40	<0.001
	Low 5	179	7	0.26	33.32	0.002
	Low 6	360	19	0.35	88.06	<0.001
	Low 7	404	33	0.55	99.95	0.047
**Zimbabwe**	High 1	374	115	1.91	77.13	<0.001
	High 2	334	91	1.68	47.90	0.001
	High 3	1281	266	1.29	94.30	0.029
	Low 1	527	38	0.43	87.80	<0.001

	Low 2	105	2	0.11	28.24	0.002
	Low 3	139	5	0.22	34.50	0.004
	Low 4	513	51	0.59	45.35	0.029
	Low 5	198	13	0.39	23.23	0.045
**Lesotho**	High 1	2880	734	1.28	72.21	<0.001
	Low 1	712	101	0.61	37.84	<0.001
	Low 2	788	129	0.71	38.24	0.034
**Guinea**	No clusters	–	–	–	–	–
**Swaziland**	No clusters	–	–	–	–	–

Supplementary Table 2 - Model assumptions in terms of parameter values

Assumption	Parameter value	Sources
HIV transmission probability per coital act per stage of infection ($p^{HIV}_{I_{\beta,i} \to S_j}$):		
Acute stage	0.0107	[22-24, 31]
Latent stage	0.0008	[2, 24]
Late stage	0.0042	[22-24]
Duration of each of HIV stages ($1/\omega_{I_\beta}$):		
Acute stage	49 days	[22, 24, 25, 27, 28, 32]
Latent stage	9.0 years	[22, 24, 25, 27, 28, 32]
Late stage	2.0 years	[22, 24, 25, 27, 28, 32]
Frequency of coital acts per HIV stage ($n_{I_{\beta,i} \leftrightarrow S_j}$):		
Acute stage	10.6 per month	[24]
Latent stage	11.0 per month	[24]
Late stage	7.1 per month	[24]
Duration of sexual partnerships ($\tau_{I_{\beta,i} \leftrightarrow S_j}$):	6 months	Representative value
Duration of the sexual lifespan ($1/\mu$)	35 years	[2, 29, 30]
Degree of assortativeness (e)	0.3	[2]
The scale parameter in the gamma distribution of the population across the risk groups (b)	2	[18]
The exponent parameter in the power law function of the distribution of sexual risk behavior (α)	1.7	[12]

References

1. Awad SF, Abu-Raddad LJ: **A signature of dramatic declines in sexual risk behavior in sub-Saharan Africa in the mid-1990s** *Under preparation* 2013.

2. Abu-Raddad LJ, Longini IM, Jr.: **No HIV stage is dominant in driving the HIV epidemic in sub-Saharan Africa.** *Aids* 2008, **22:**1055-1061.

3. Abu-Raddad LJ, Patnaik P, Kublin JG: **Dual infection with HIV and malaria fuels the spread of both diseases in sub-Saharan Africa.** *Science* 2006, **314:**1603-1606.

4. Kretzschmar M, Morris M: **Measures of concurrency in networks and the spread of infectious disease.** *Math Biosci* 1996, **133:**165-195.

5. Morris M: **Sexual networks and HIV.** *AIDS* 1997, **11:**S209-S216.

6. Watts CH, May RM: **The influence of concurrent partnerships on the dynamics of HIV/AIDS.** *Math Biosci* 1992, **108:**89-104.

7. Kretzschmar M, Morris M: **Measures of concurrency in networks and the spread of infectious disease.** *Math Biosci* 1996, **133:**165-195.

8. Morris M: **Sexual networks and HIV.** *AIDS* 1997, **11 Suppl A:**S209-216.

9. May RM, Anderson RM: **The Transmission Dynamics of Human Immunodeficiency Virus (Hiv).** *Philosophical Transactions of the Royal Society of London Series B-Biological Sciences* 1988, **321:**565-607.

10. Ferry B, Carael M, Buve A, Auvert B, Laourou M, Kanhonou L, de Loenzien M, Akam E, Chege J, Kaona F: **Comparison of key parameters of sexual behaviour in four African urban populations with different levels of HIV infection.** *Aids* 2001, **15 Suppl 4:**S41-50.

11. Lagarde E, Auvert B, Carael M, Laourou M, Ferry B, Akam E, Sukwa T, Morison L, Maury B, Chege J, et al: **Concurrent sexual partnerships and HIV prevalence in five urban communities of sub-Saharan Africa.** *Aids* 2001, **15:**877-884.

12. Awad SF, Cuadros DF, Abu-Raddad LJ: **Generic patterns of HIV infection distribution in human populations.** *Under preparation* 2013.

13. Barrat A, Barthelemy M, Pastor-Satorras R, Vespignani A: **The architecture of complex weighted networks.** *Proceedings of the National Academy of Sciences of the United States of America* 2004, **101:**3747-3752.

14. Boccaletti S, Latora V, Moreno Y, Chavez M, Hwang DU: **Complex networks: Structure and dynamics.** *Physics Reports* 2006, **424:**175-308.

15. Watts DJ, Strogatz SH: **Collective dynamics of 'small-world' networks.** *Nature* 1998, **393:**440-442.

16. Barabási AL: *Linked: how everything is connected to everything else and what it means for business, science and everyday life.* London: First Plume Printing; 2003.

17. Garnett GP, Anderson RM: **Factors controlling the spread of HIV in heterosexual communities in developing countries: patterns of mixing between different age and sexual activity classes.** *Philosophical transactions of the Royal Society of London Series B, Biological sciences* 1993, **342:**137-159.

18. Cuadros DF, Crowley PH, Augustine B, Stewart SL, Garcia-Ramos G: **Effect of variable transmission rate on the dynamics of HIV in sub-Saharan Africa.** *BMC infectious diseases* 2011, **11:**216.

19. Handcock MS, Jones JH: **Likelihood-based inference for stochastic models of sexual network formation.** *Theoretical population biology* 2004, **65:**413-422.

20. Hamilton DT, Handcock MS, Morris M: **Degree distributions in sexual networks: a framework for evaluating evidence.** *Sex Transm Dis* 2008, **35:**30-40.

21. Bansal S, Grenfell BT, Meyers LA: **When individual behaviour matters: homogeneous and network models in epidemiology.** *Journal of the Royal Society, Interface / the Royal Society* 2007, **4:**879-891.

22. Pinkerton SD: **Probability of HIV transmission during acute infection in Rakai, Uganda.** *AIDS Behav* 2008, **12:**677-684.

23. Hollingsworth TD, Anderson RM, Fraser C: **HIV-1 transmission, by stage of infection.** *The Journal of infectious diseases* 2008, **198:**687-693.

24. Wawer MJ, Gray RH, Sewankambo NK, Serwadda D, Li X, Laeyendecker O, Kiwanuka N, Kigozi G, Kiddugavu M, Lutalo T, et al: **Rates of HIV-1 transmission per coital act, by stage of HIV-1 infection, in Rakai, Uganda.** *The Journal of infectious diseases* 2005, **191:**1403-1409.

25. UNAIDS: **UNAIDS Reference Group on Estimates, Modelling and Projections.** 2007.

26. UNAIDS/WHO: ***AIDS epidemic update 2007.*** In *Book AIDS epidemic update 2007*. City.

27. UNAIDS/WHO: ***AIDS epidemic update 2010: UNAIDS fact sheet* (available at http://www.unaids.org/documents/20101123_FS_SSA_em_en.pdf, accessed 23 July 2012).** In *Book AIDS epidemic update 2010: UNAIDS fact sheet (available at http://www.unaids.org/documents/20101123_FS_SSA_em_en.pdf, accessed 23 July 2012)*. City; 2010.

28. UNAIDS/WHO: **Epidemiological data, HIV estimates 1990-2009 (available at http://www.unaids.org/en/dataanalysis/epidemiology/).** In *Book Epidemiological data, HIV estimates 1990-2009 (available at http://www.unaids.org/en/dataanalysis/epidemiology/)*. City; 2010.

29. Buve A, Carael M, Hayes RJ, Auvert B, Ferry B, Robinson NJ, Anagonou S, Kanhonou L, Laourou M, Abega S, et al: **Multicentre study on factors determining differences in rate of spread of HIV in sub-Saharan Africa: methods and prevalence of HIV infection.** *Aids* 2001, **15 Suppl 4:**S5-14.

30. UNAIDS: **UNAIDS Report on the Global AIDS Epidemic 2010.** In *Book UNAIDS Report on the Global AIDS Epidemic 2010*. City; 2010.

31. Wawer MJ, Reynolds SJ, Serwadda D, Kigozi G, Kiwanuka N, Gray RH: **Might male circumcision be more protective against HIV in the highly exposed? An immunological hypothesis.** *Aids* 2005, **19:**2181-2182.

32. UNAIDS/WHO: **AIDS epidemic update 2007.** 2007.
